# Effects and determinants of tuberculosis drug stockouts in South Africa

**DOI:** 10.1186/s12913-019-3972-x

**Published:** 2019-04-03

**Authors:** L. E. M. Koomen, R. Burger, E. K. A. van Doorslaer

**Affiliations:** 1 Erasmus School of Health Policy and Management, Erasmus University Rotterdam, Burgemeester Oudlaan 50, Rotterdam, PA 3062 The Netherlands; 2 Department of Economics, Stellenbosch University, Stellenbosch, South Africa; 3 Erasmus School of Economics, Erasmus University Rotterdam, Burgemeester Oudlaan 50, Rotterdam, PA 3062 The Netherlands

**Keywords:** Drug stockouts, Tuberculosis, Treatment outcomes, Equity

## Abstract

**Background:**

The frequent occurrence of medicine stockouts represents a significant obstacle to tuberculosis control in South Africa. Stockouts can lead to treatment alterations or interruptions, which can impact treatment outcomes. This study investigates the determinants and effects of TB drug stockouts and whether poorer districts are disproportionately affected.

**Methods:**

TB stockout data, health system indicators and TB treatment outcomes at the district level were extracted from the *District Health Barometer* for the years 2011, 2012 and 2013. Poverty terciles were constructed using the Census 2011 data to investigate whether stockouts and poor treatment outcomes were more prevalent in more impoverished districts. Fixed-effects regressions were used to estimate the effects of TB stockouts on TB treatment outcomes.

**Results:**

TB stockouts occurred in all provinces but varied across provinces and years. Regression analysis showed a significant association between district per capita income and stockouts: a 10% rise in income was associated with an 8.50% decline in stockout proportions. In terms of consequences, after controlling for unobserved time invariant heterogeneity between districts, a 10% rise in TB drug stockouts was found to lower the cure rate by 2.10% (*p* < 0.01) and the success rate by 1.42% (*p* < 0.01). These effects were found to be larger in poorer districts.

**Conclusions:**

The unequal spread of TB drug stockouts adds to the socioeconomic inequality in TB outcomes. Not only are stockouts more prevalent in poorer parts of South Africa, they also have a more severe impact on TB treatment outcomes in poorer districts. This suggests that efforts to cut back TB drug stockouts would not only improve TB treatment outcomes on average, they are also likely to improve equity because a disproportionate share of this burden is currently borne by the poorer districts.

**Electronic supplementary material:**

The online version of this article (10.1186/s12913-019-3972-x) contains supplementary material, which is available to authorized users.

## Background

Medicine shortages affect countries worldwide, but in particular developing countries [1–4] The evidence to date is limited but suggests that stockouts are associated with treatment alteration and discontinuation, and with worse health outcomes, but none of these studies provides any estimates of the impact of stockouts on health outcomes. It also does not examine whether stockouts affect rich and poor communities differentially.

The drug stockout problem is particularly pressing in South Africa for TB medicines for four reasons. First, South Africa is facing a huge TB epidemic, with 438,000 cases a year and an annual mortality of 123,000 people [5]. Secondly, South Africa has very high levels of drug stockouts with 14% of the primary health care facilities reporting a TB drug stockout in 2011 [6]. Thirdly, TB is a lethal, but treatable disease and drugs are dispensed for free to TB patients by the public health clinics, but periods of stockouts during the six-month treatment period could have a severe adverse impact on treatment success. Finally, the country has notoriously high levels of income inequality [7] which makes it likely that the drug stockouts do not affect richer and poorer districts equally.

### Drug stockouts in South Africa

South Africa’s pharmaceutical chain consists of several links. The National Department of Health is responsible for contracting with pharmaceutical companies. Every two years, the government releases and awards tenders for TB drugs. The Provincial Departments of Health take care of the planning, funding, monitoring and evaluation of pharmaceutical services in the provinces. The Provincial Medicine Depots are responsible for procurement and storage of the drugs, and for their delivery to district pharmacies and health facilities. Health facilities can order medicines at the Provincial Medicine Depot or at district pharmacies [8].

A performance audit of the management of pharmaceuticals in South Africa revealed that problems occur at many points along the pharmaceutical chain [8]. Potential reasons for stockouts at the National and Provincial Departments of Health are late drug tenders; essential medicines not provided in government tenders; lack of policies to manage pharmaceuticals; inadequate budgets for the population’s health needs; and, lack of oversight [8]. Causes of drug stockouts at the pharmaceutical companies are sudden unforecasted increases in demand (e.g. because of guideline changes without sufficient preparation); shortages of active ingredients; manufacturing issues; and, the limited number of pharmaceutical companies due to mergers and acquisitions [4, 8–10]. The relatively small number of pharmaceutical companies makes the pharmaceutical chain vulnerable to unexpected events [11]. In case of a shortage at the supplier, the supplier will first deliver to provinces that impose penalties for late delivery. Only two of the nine provinces impose such penalties (Gauteng and the Western Cape) [8].

Causes of drug stockouts in the medicine depots and health facilities include damage, expiry and theft due to poor storage and stock management. Other causes are a lack of resources - which can result in late or non-payment of pharmaceutical companies; lack of skilled staff; unexpected demand due to unpredictable migration patterns; poor infrastructure, theft and corruption [12]; and, inadequate stock monitoring [2, 4, 8].

Appropriate drug stock management at the Provincial Department of Health, Provincial Medicine Depots and health facilities requires a comprehensive monitoring system, skilled staff and good infrastructure. There are several reasons why stockouts may affect poor districts more. First, poorer districts often struggle to attract skilled and experienced staff and have worse infrastructure [13]. Secondly, remote areas are more likely to be poorer, which generates additional demands on resources and logistics to deliver drugs to these locations. Finally, the two most affluent provinces in South Africa (Western Cape and Gauteng) often get preferential treatment from suppliers because they impose penalties for late delivery.

Regarding the consequences of drug stockouts, several other studies in sub-Saharan Africa have suggested that medicine shortages may affect treatment and treatment outcomes [6, 14, 15]. A study in Malawi showed that one of the reasons for changing the HIV treatment regimen was drug stockouts [14]. A study in Ivory Coast found that stockouts caused 9% of the HIV treatment discontinuations and 30% of the treatment alterations. Patients who interrupted treatment because of stockouts had an increased probability of dying in comparison to patients who continued treatment [15]. Seunanden and Day [6] found that TB drug stockout rates in South Africa were associated with higher death rates in 2011.

These findings for developing countries are in line with findings from studies on drug stockouts conducted in developed countries [10, 16–18]. In the United States shortage of the injectable anaesthetic drug propofol caused infection risk for 40,000 patients due to mistakes with a different dosage of the drug [16]. Moreover, lower event-free survival rates were seen in children with cancer treated on a different regimen due to drug stockouts [17]. Drug stockouts may also have an impact on costs because they increase the workload for health staff, as alternative treatments have to be found. Alternative drugs can also be more costly [18]. This has economic consequences: A survey conducted in the United States showed that approximately US $216 million was spent to manage drug stockouts on an annual basis [10].

Drug stockouts can also impede treatment adherence [19, 20]. Strict treatment adherence is crucial for successful TB treatment [21–23] (Additional file 1: Box 1). This knowledge and existing literature on drug stockouts, lead us to expect that TB drug stockouts have a negative impact on TB treatment outcomes. This negative impact has not yet been demonstrated in other work, like [6] who reported a positive correlation between TB drug stockouts and TB deaths, but without further control for confounding due to observed and unobserved factors. In our study we adopted a fixed-effects regression approach to better control for possible confounding factors and examined the heterogeneity of health effects by district poverty. We also focused on the public primary care sector, since this is where most cases of TB are treated in South Africa [21].

## Methods

We use two data sources that were linked at the district level. *The District Health Barometer* [24] was used to extract TB drug stockout data, TB health outcomes and health system characteristics. This dataset contains annually aggregated indicators of public primary health facilities in South Africa for the years 2011, 2012 and 2013 at the district level. Socioeconomic information was obtained from the *Census survey 2011* [25] household data and then aggregated at the district level. Variable definitions and descriptive statistics can be found in Tables 1 and 2. Of the 52 districts in South Africa 51 were included, as stockout proportions were missing for Tshwane District. Stockout proportions were also missing for Eden and Overberg in 2012 and for Central Karoo and Cape Town in 2011 (all located in the Western Cape Province).

**Table 1:** Definition of the indicators

Indicator	Definition
TB stockout proportion	Annual proportion of primary health care facilities reporting at least one TB drug stockout. Facilities report a stockout when any of the TB drugs are out of stock. Length of stockout duration is not reported. The numerator of this proportion is all facilities that reported a stockout. The denominator is the number of reporting facilities.
TB cure rate	Percentage of *new* smear-positive TB patients who were smear negative in the last month of their treatment, and at least one time before.
TB success rate	Percentage of *all* TB *patients on treatment* (smear positive, smear negative, extra-pulmonary patients) who were cured (smear negative) or *completed* treatment (not cured, but finished treatment).
TB death rate	Percentage of all TB *patients on treatment* who died
TB incidence	Number of TB *patients on treatment* per 100,000 inhabitants
TB/HIV coinfection	Percentage of TB *patients on treatment* who are infected with HIV
Expenditure/visit	Provincial and local government expenditure in South African Rand on primary health care per visit for the non-privately insured population
District income	Average income in South African Rand per capita in a district
Urban	Proportion of people in a district who live in a city

TB indicators and expenditure/visit extracted from District Health Barometer [24]. District income and urban extracted from Census 2011 [25]

**Table 2:** Descriptive statistics

Indicator	Mean	SD	Min	Max
TB stockout proportion	10.49	8.76	0.10	44.30
TB cure rate	74.58	8.14	47.92	95.68
TB success rate	74.66	7.03	46.74	90.12
TB death rate	9.18	3.27	2.78	16.93
TB incidence	774.21	262.07	285.04	1511.92
TB/HIV coinfection	86.79	7.73	49.53	98.45
Expenditure/visit	301.51	51.74	175.32	469.13
District income	50,032	22,530	19,535	121,421
Urban	0.59	0.32	0.05	1.00

*SD* = standard deviation, *Min* = minimum value, *Max* = maximum value

### Statistical analysis

First, we describe differences in average TB drug stockouts and TB treatment outcomes by district between poverty categories.^1^ Districts were divided into three poverty categories: districts with lowest poverty (poverty rate 26–35%), districts with intermediate poverty (poverty rate 35–42%), and districts with highest poverty (poverty rate 42–52%). Average values of stockouts and treatment outcomes were compared with a one-way ANOVA.

To investigate potential determinants of TB drug stockout proportions per district, we ran a weighted regression analysis on a number of covariates. Mean per capita household income and degree of urbanisation of a district were used as proxies for district socioeconomic status. Provincial and local government spending per health visit was included as a health system characteristic. A dummy variable was included for the district with the provincial medicine depot location. Due to the skewed distribution of district income, log transformation was applied to the income variable. Time effects and province effects are captured by a year and province dummy, and observations were weighted based on the number of TB patients on treatment to give greater weight to districts with larger volumes of TB patients on treatment.

To examine the effect of TB stockouts on three treatment outcomes by district, i.e. TB cure rate, TB success rate and TB death rate, we used a regression with time and district fixed effects. This method not only exploits the time variation *between* years but also *within* districts to identify effects on TB outcomes. No lagged effects were included because all treatment outcomes are short-term effects; the duration of standard TB treatment is six months [21] and most TB deaths occur in the first months of treatment [26]. As the fixed-effect regression has the advantage that it controls for all time invariant unobservable district factors, most of the potential omitted variable bias is avoided. This is important, because there might be specific unobservable district characteristics that affect both the drug stockout proportion and the treatment outcomes.^2^ Moreover, we also control for two time varying factors: TB incidence and TB/HIV co-infection which have been shown to affect TB treatment outcomes negatively [27, 28]. Due to the high HIV-TB coinfection rates and the increase in ART coverage over this period, we also include an estimate of ART coverage in the regressions. The ART coverage rate for each district was estimated as the number of patients on ART as share of the population.

Under the assumption that were no other unobserved factors that varied over time together with the stockouts, we interpret the estimated coefficient of the stockout variable as a causal effect**.** The analysis was performed for the whole sample and separately for each poverty category. Statistical significance testing of differences in health effects between poverty groupings was done using interactions between the stockout proportion and the poverty category.

We also performed robustness check by adding patients who were classified as lost to follow to the district's TB deaths. This was done because the TB death rate might be an underestimation of the TB deaths because it misses TB cases that were lost to follow-up prior to their deaths^3^ (Additional file 1: Table S9).

## Results

**Fig. 1:**
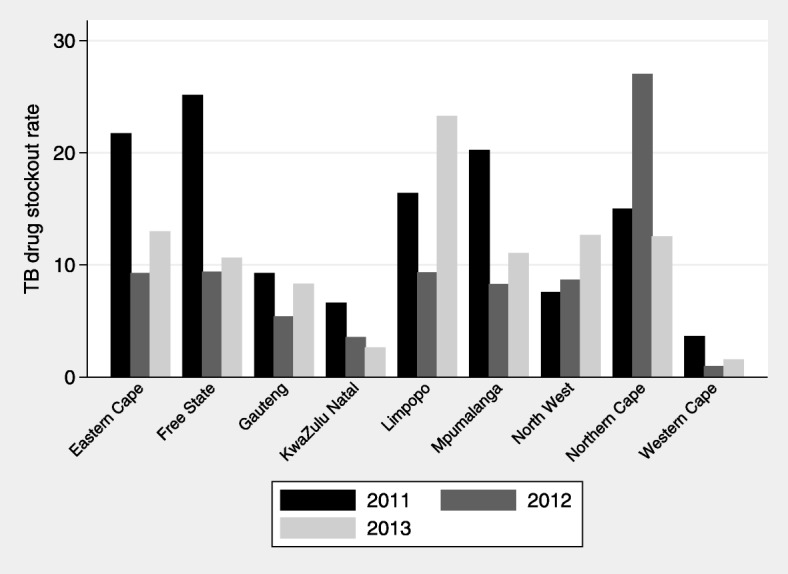
Average district TB stockout proportions, by province in 2011, 2012 and 2011. Rates are weighted by number of patients on TB treatment

There is substantial variation in TB stockout proportions across provinces and across the years 2011, 2012 and 2013 (Fig. 1). In our study period, the Western Cape was on average the province with the lowest and the Northern Cape the province with the highest stockout proportions (3.84 vs. 16.52).

Table 3 lists the average TB stockout proportions by district and TB treatment outcomes by district by poverty categories. TB stockout proportions are higher and TB treatment outcomes are seen to be worse in districts with higher poverty. The ANOVA confirms that TB outcomes vary by level of poverty of the district.

**Table 3:** Mean TB stockout proportions and TB treatment outcomes by district poverty category

Poverty category	TB drug stockouts	TB cure rate	TB success rate	TB death rate
Least poor	9.496	76.04	76.66	7.894
	(1.280)	(0.816)	(0.735)	(0.506)
Middle	9.229	75.05	74.36	9.054
	(1.081)	(1.301)	(1.207)	(0.457)
Poorest	12.67	72.20	72.65	10.66
	(1.324)	(1.198)	(0.902)	(0.323)
*P* value ANOVA				
Least poor vs. middle	0.82	0.50	< 0.01	< 0.05
Least poor vs. poorest	< 0.01	< 0.01	< 0.01	< 0.01
Middle vs. poorest	< 0.05	< 0.01	0.16	< 0.01

Percentage of people living in poverty in the least poor districts: 26–35%, middle: 35–42%, poorest: 42–52%. Tested with one-way ANOVA. Standard error in parentheses

### What determines stockout proportions?

The regression analysis for 2011–2013 shows that district per capita income is negatively associated with stockouts (Table 4). Ceteris paribus, a 10% rise in income (log transformed income) is associated with an 8.5% decline in TB stockout proportions. Having the Provincial Medicine Depot located in the district is associated with higher stockout proportions (*p*-value < 0.05). Provincial and local government expenditure per visit and degree of urbanisation are not correlated with TB stockouts.

**Table 4:** Weighted least squares (WLS) regression results determinants of TB drug stockouts

Variables	WLS ‘11-‘13
	TB drug stockouts
Log income	−8.499**
	(3.273)
Expenditure/visit	−0.00881
	(0.0137)
Urban	2.266
	(3.836)
Provincial Medicine Depot	3.210**
	(1.356)
Constant	106.9***
	(32.48)
Observations	149
R-squared	0.599

Standard errors in parentheses

***p < 0.01, **p < 0.05, *p < 0.1

Time and province effects are captured by year and province dummies. Districts were weighted based on the number of TB patients on treatment to give greater weight to districts with larger volumes of TB patients on treatment

### Effects of stockouts on TB treatment outcomes

**Fig. 2:**
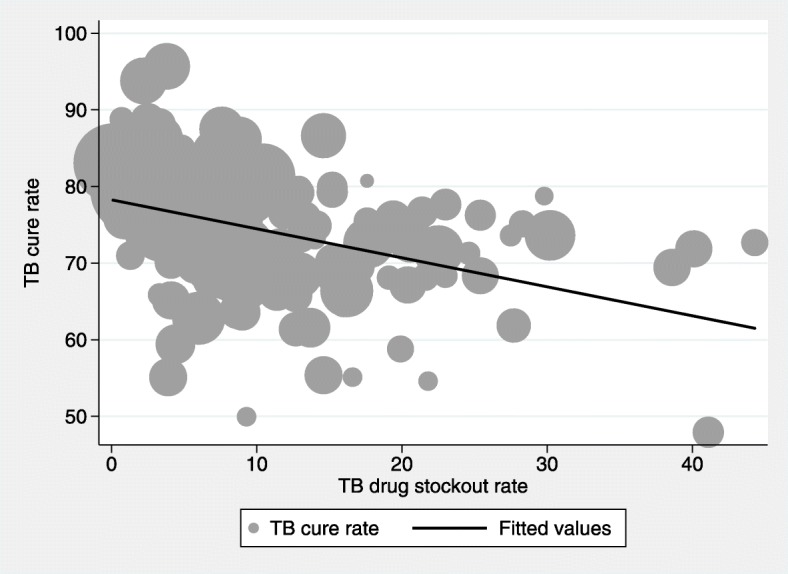
Association between TB cure rate and TB drug stockouts. Fitted line is weighted by number of people on TB treatment (shown by dot size)

**Fig. 3:**
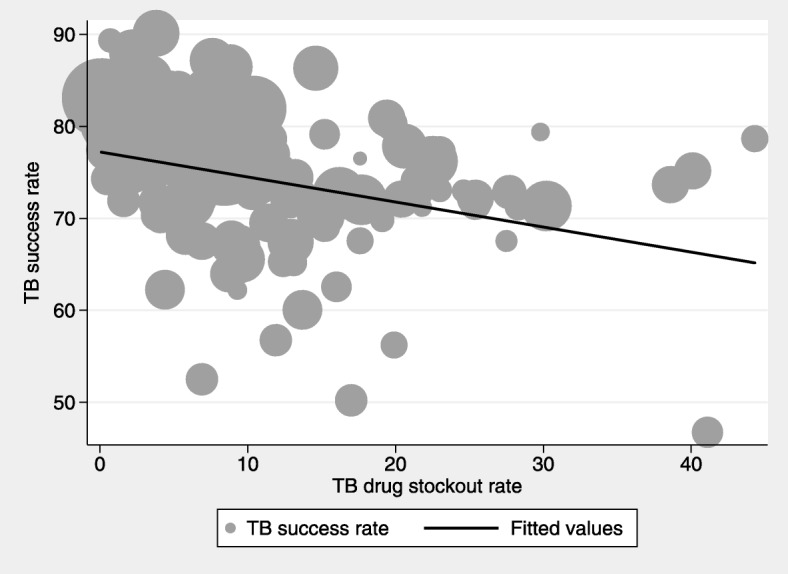
Association between TB success rates and TB drug stockouts. Fitted line is weighted by number of people on TB treatment (shown by dot size)

**Fig. 4:**
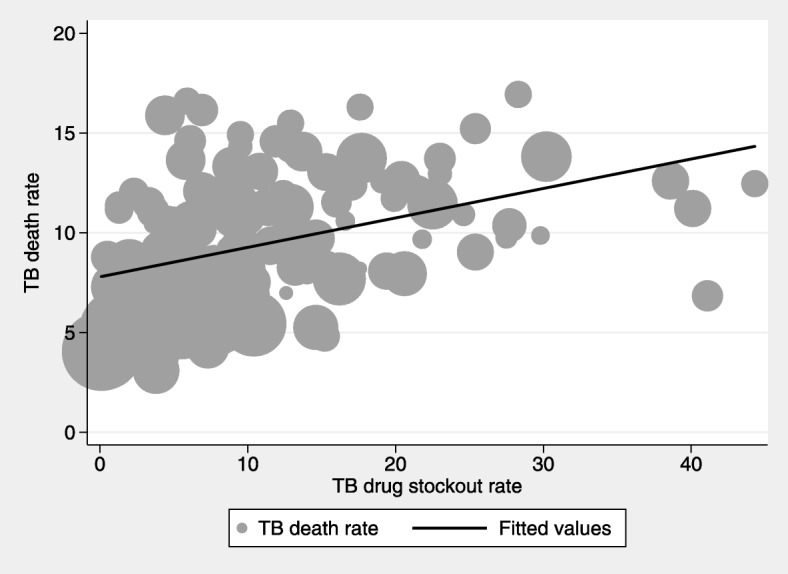
Association between TB death rates and TB drug stockouts. Fitted line is weighted by number of people on TB treatment (shown by dot size)

Figures 2, 3 and 4 show graphical plots of the association between TB treatment outcomes and TB drug stockouts at the district level. While there appears to be a negative correlation between TB cure rates, TB success rates and TB drug stockouts (Figs. 2 and 3), a positive correlation is observed between TB death rates and TB drug stockouts (Fig. 4). This suggests that, in general, districts with more TB drug stockouts are likely to experience worse TB treatment outcomes.

The regression analysis for all districts demonstrates that TB drug stockouts have a significant negative effect on TB cure rates and TB success rates (Tables 5 and 6). It suggests that, in the period considered, a 10% rise in TB drug stockout proportions resulted in a 2.14% decline of the TB cure rate and a 1.43% decline of the TB treatment success rate. Tables 5, 6 and 7 show that the negative consequences of TB drug stockouts were largest in the poorest districts. In those, a 10% increase in TB drug stockout proportion led to a 3.25% decline in the TB cure rate and a 2.78% decline in the TB success rate. In the districts with the lowest poverty rates, we did not find any significant effect of TB drug stockouts on TB treatment outcomes.

**Table 5:** Regression results for TB cure rate

Variables	TB cure rate	TB cure rate	TB cure rate	TB cure rate
Poverty category	All	Least poor	Middle	Poorest
TB drug stockout proportion	−.214***	.048	−.254**	−.325**
	(.069)	(.115)	(.096)	(.124)
Conf interval	−.351/−.076	−.1890/.285	−.452/ -.057	−.578/ -.072
Constant	81.237***	77.200***	64.862***	125.070***
	(12.731)	(26.083)	(17.560)	(23.090)
Conf interval	55.952/106.523	23.107/131.293	28.894/100.830	77.914/172.227
District FEs	Yes	Yes	Yes	Yes
Year FEs	Yes	Yes	Yes	Yes
Observations	149	44	51	54
Number of districts	51	16	17	18
R-squared	0.049	0.095	0.409	0.045

Standard errors in parentheses

***p < 0.01, **p < 0.05, *p < 0.1

Note: Analysis was controlled for TB/HIV coinfection, TB incidence and ART coverage. Districts were weighted based on the number of TB patients on treatment

**Table 6:** Regression results for TB success rate

Variables	TB success rate	TB success rate	TB success rate	TB success rate
Poverty category	All	Least poor	Middle	Poorest
TB drug stockout proportion	−.143***	.070	−.160**	−.278**
	(.054)	(.083)	(.070)	(.103)
Conf interval	−.250/.037	−.102/.242	−.303/−.017	−.489/−.067
Constant	82.203***	91.210***	68.765***	101.187***
	(9.915)	(18.872)	(12.722)	(19.318)
Conf interval	62.512/101.895	52.072/130.348	42.705/94.825	61.734/140.638
District FEs	Yes	Yes	Yes	Yes
Year FEs	Yes	Yes	Yes	Yes
Number of districts	51	16	17	18
R-squared	0.003	0.152	0.290	0.047

Standard errors in parentheses

***p < 0.01, **p < 0.05, *p < 0.1

Note: Analysis was controlled for TB/HIV coinfection, TB incidence and ART coverage. Districts were weighted based on the number of TB patients on treatment

**Table 7:** Regression results for TB death rate

Variables	TB death rate	TB death rate	TB death rate	TB death rate
Poverty category	All	Least poor	Middle	Poorest
TB drug stockout proportion	−.009	.001	.046*	−.049**
	(.014)	(.031)	(.027)	(.022)
Conf interval	−.037/.019	−.064/.066	−.009/.101	−.093/−.004
Constant	12.390***	4.930	13.642***	13.804***
	(2.616)	(7.158)	(4.880)	(4.052)
Conf interval	7.195/17.585	−9.914/19.774	3.647/23.638	5.529/22.078
District FEs	Yes	Yes	Yes	Yes
Year FEs	Yes	Yes	Yes	Yes
Number of districts	51	16	17	18
R-squared	0.004	0.182	0.226	0.053

Standard errors in parentheses

***p < 0.01, **p < 0.05, *p < 0.1

Note: Analysis was controlled for TB/HIV coinfection, TB incidence and ART coverage. Districts were weighted based on the number of TB patients on treatment

The results for death rates are more surprising. Overall, we do not find any effect on death rates. But for the districts in the middle category we found that an increase of 10% in districts stockouts results in an increase of 0,46% of deaths. For the poorest districts, the result is more surprising. The coefficient is negative and significantly different from zero at the 5% level of significance. However, we are concerned that this counterintuitive result may be attributable to the underestimation of the TB death rate because it misses TB cases that were lost to follow-up prior to their deaths^3^. We do not find any significant effect of TB drug stockouts on this combined outcome measure of TB deaths and patients lost to follow up.

## Discussion

Our study is the first that has tried to exploit the variation in drug stockouts across districts and over time in South Africa to examine the determinants and the effects of TB drug stockouts.

First, we found that TB drug stockouts are more prevalent in districts with lower income: a 10% rise in income was associated with an 8.5% decline in TB drug stockouts. This suggests that poorer districts are likely to suffer a greater burden of stockouts.

Secondly, and contrary to a priori expectation, we found that districts in which the Provincial Medicine Depot was located were more likely to experience a TB drug stockout. This suggests that greater distance to a Provincial Medicine Depot does not appear to operate as a constraint on adequate stock levels in South Africa. It also suggests that facilities located in the same district as the provincial depot overly reliant on the provincial depot.

Third, after controlling for time invariant unobservables, we estimate that TB stockouts had substantial negative effects on TB cure and success rates by district. Our results indicate that, on average, a 10% rise in the TB drug stockout proportion results in a 2.14% decline in TB cure rate and a 1.43% decline in TB success rate. These negative consequences of TB drug stockouts were larger in the districts with higher poverty rates. This confirms that the negative effects are primarily concentrated in the poorest districts of South Africa and are likely to further exacerbate the socioeconomic inequalities in TB treatment outcomes.

Our results imply that TB drug stockouts can limit the effective control of the TB epidemic, especially in districts with higher rates of poverty. This is because, when fewer patients are cured, more patients remain infectious and are able to spread TB. Improved reliability of the TB drug supply is therefore essential to enhance the effectiveness of TB epidemic control.

The finding that TB drug stockouts seem to have a greater negative impact on TB cure rates than on TB success rates deserves further discussion. The TB cure rate measures the fraction of all smear-positive TB patients who are smear negative in the last two months of their treatment. The TB success rate measures the fraction of all TB patients who completed their treatment and all smear-positive TB patients who are smear negative in the last two months of their treatment.

The South African public health system transitioned to Genexpert^4^ in 2012 which means that a small minority of patients receive smear tests. For patients who did not receive smear tests the success rate merely captures whether they have had a recent clinic visit to collect their treatment. Patients included in the TB success rate are therefore not necessarily cured. For TB to be cured, the treatment regimen must be followed strictly and interruptions can have serious consequences for cure [21–23], whilst for TB success treatment must be completed. Interruptions may therefore have less impact, as reflected in our results.

The finding of a negative impact of TB drug stockouts and TB death rates in the poorest districts is counterintuitive, but the further analysis with lost to follow up patients suggests that the initial finding may be attributable to missed deaths and the higher concentration of such missed deaths in poorer districts.

We found that the effects of TB drug stockouts are worse in districts with more people living in poverty. This could be due to the fact that in these districts patients have fewer options to look for alternatives to access TB drugs. When a health facility has a stockout, patients can go to another health facility to collect their TB drugs. This requires extra travel and involves time and money costs. Another option would be to buy the TB medicine at a private pharmacy or clinic. When these options are not feasible for cash-constrained patients, they are forced to interrupt TB treatment until the TB drugs are available.

TB medicines were not the only drugs that have had stockout problems in South Africa. Stockout surveys [29–31] have shown that many other essential medicines were also affected. This problem was acknowledged by the National Department of Health and some measures have been taken to curb the problem [32]. However, and in spite of these, 20% of health facilities in South Africa still reported stockouts of essential medicines (including TB drugs) in 2016 [33].

Since the consequences of TB drug stockouts for TB treatment outcomes are severe, emphasis should be placed on further strengthening of the pharmaceutical chain in South Africa. A short-term solution to ensure continuous drug supply to TB patients could be a public-private partnership in which TB patients can collect their TB drugs for free at a private pharmacy or -clinic in the case of a stockout in the public health facility.^5^

While contributing to the literature on the subject, our study also has limitations. First, the aggregated annual stockout proportion at the district level is measured with substantial imprecision. The district stockout proportion was not weighted by clinic size and does not include any information about the duration of the stockout. As a result, a stockout of a few days is weighted equally to a stockout of a month. Since the publicly available data were already aggregated and not available at the facility level, it was not possible to adjust and refine these rates. Secondly, as our analysis was performed at the district level, it may suffer from ecological fallacy or bias. Some districts are quite large and considerable variation in TB drug stockout proportion and TB treatment outcomes between clinics within districts exist. TB drug stockouts can occur in one clinic, whereas bad TB treatment outcomes can occur in another clinic. These differences disappear when TB drug stockout proportion and TB treatment outcomes are averaged on a district level. A potential future analysis at facility level could solve this problem but will run into another problem: that of attributing TB patients to clinics. Last but not least, the fixed-effect regression analysis controlled for time invariant unobservables, TB incidence and HIV coinfection. To the extent that any other relevant time varying unobservables were omitted, our estimates may still be subject to some omitted variable bias.

## Conclusion

The persistence of TB drug stockouts in South Africa not only hampers the effective control of TB but its unequal occurrence across districts also adds to the socioeconomic inequality in TB outcomes. We find that stockouts are not only more prevalent in poorer parts of South Africa, they also have greater detrimental effects on the TB treatment outcomes of the poorer districts. This suggests that efforts to reduce TB drug stockouts are essential in the fight against the TB burden and its impact on socioeconomic inequality.

## Additional file

Additional file 1: **Box 1.** Background information on tuberculosis treatment in South Africa. **Table S8.** Districts divided in poverty categories: An overview of the South African districts divided into poverty categories. **Table S9.** Regression results for TB death rate + lost to follow up (DLTFU): The results of the regression analysis for TB stockouts on TB death rate including lost to follow up patients. (DOCX 29 kb)

## Abbreviations

ART: Antiretroviral therapy
Conf interval: Confidence interval
DLTFU: Death rate + lost to follow up
FE: Fixed effects
HIV: Human immunodeficiency virus
Max: Maximum value
Min: Minimum value
SD: Standard deviation
TB: Tuberculosis
WLS: Weighted least squares

## Footnotes

1 A poverty threshold of R575 (South African Rand) per month per household was used. This is an inflation adjusted lower-bound poverty line, estimated with the cost-for-basic needs poverty line suggested by Özler [34, 35]). Table 8 in the Appendix lists the districts by poverty category.

2 An example of a potential unobserved district characteristic is ‘bad governance’. This can influence TB drug stockouts, because stock management might be inadequate. And it can influence TB treatment outcomes, because TB treatment guidelines might not be implemented in health facilities.

3 See Health Systems Trust [36].

4 Genexpert is a molecular TB test detecting the presence of TB bacteria. Its main advantages are that it can give a result in less than two hours (accurate culture tests can take weeks); that it is more sensitive than sputum microscopy for HIV-positive patients; and, that it also tests for resistance to the drug Rifampicin. It was endorsed by the WHO in 2010. One of the main downsides is that it cannot be used to monitor treatment progress given that it detects both live and dead bacteria.

5 For an example of such an experiment, see e.g. the Central Chronic Medicine Dispensing and Distribution Programme (CCMDD) available at http://www.hst.org.za/hstconference/hstconference2016/Presentations/roma_ramphal_hst_conf_final_04.05.2016.pdf.
