# Supplementary material for: Effects and determinants of tuberculosis drug stockouts in South Africa
Source: BMC Health Serv Res. 2019 Apr 3;19:213. doi: 10.1186/s12913-019-3972-x (PMC6448237; doi:10.1186/s12913-019-3972-x)
Supplement: Supplementary file 1 — Box 1. Background information on tuberculosis treatment in South Africa. Table S8. Districts divided in poverty categories: An overview of the South African districts divided into poverty categories. Table S9. Regression results for TB death rate + lost to follow up (DLTFU): The results of the regression analysis for TB stockouts on TB death rate including lost to follow up patients. (DOCX 29 kb) [file 12913_2019_3972_MOESM1_ESM.docx]

**APPENDIX**

**Contains:**

- **Box 1:** Background information on tuberculosis treatment in South Africa.
- **Table 8. Districts divided in poverty categories:** An overview of the South African districts divided into poverty categories.
- **Table 9. Regression results for TB death rate + lost to follow up (DLTFU)**: The results of the regression analysis for TB stockouts on TB death rate including lost to follow up patients.

| **BACKGROUND ON TUBERCULOSIS TREATMENT IN SOUTH AFRICA**  89% of TB patients in South Africa are infected with pulmonary TB [1]. These patients are infectious when their sputum contains tuberculosis bacilli. TB can then be spread through the air when a patient coughs or talks. Tuberculosis bacilli can be found in the sputum with a sputum smear. A patient is much less infectious when the sputum does not contain tuberculosis bacilli (sputum smear negative). The standard treatment regimen for (pulmonary) TB consists of an intensive two-month phase and a four-month continuation phase. In the first phase four different antibiotics are given, followed by two antibiotics in the continuation phase. TB treatment is initiated and monitored by a TB nurse [2].  Health care and medicines in the public sector are free of charge for all patients [3]. |
| --- |

**Box 1.** Background on tuberculosis treatment in South Africa

*References:*

*1. World Health Organization (WHO). Global tuberculosis report. Vol. 69, Pharmacological Reports. 2018. 1-277 p.*

*2. TB DOTS Strategy Coordination. National Tuberculosis Management Guidelines 2014 [Internet]. 2014. 19-28 p. Available from: http://www.sahivsoc.org/upload/documents/NTCP_Adult_TB Guidelines 27.5.2014.pdf*

*3. Soul City Research Unit. Literature Review of TB in South Africa [Internet]. 2015. Available from: https://www.soulcity.org.za/projects/tuberculosis/research/literature-review-on-tuberculosis-in-south-africa.pdf*

| **Table 8. Districts divided in poverty categories** | | | | | |
| --- | --- | --- | --- | --- | --- |
| **Districts with lowest poverty rates** | | **Districts with intermediate poverty rates** | | **Districts with highest poverty rates** | |
| **District name** | **Province** | **District name** | **Province** | **District name** | **Province** |
| Cape Winelands | Western Cape | Xhariep | Free State | T. Mofutsanyana | Free State |
| Cape Town | Western Cape | Jehweleputswa | Free State | A. Nzo | Eastern Cape |
| Central Karoo | Western Cape | Buffalo City | Eastern Cape | Amathole | Eastern Cape |
| Eden | Western Cape | N. Mandela Bay | Eastern Cape | C. Hani | Eastern Cape |
| West Coast | Western Cape | Ekurhuleni | Gauteng | Joe Gqabi | Eastern Cape |
| Overberg | Western Cape | Sedibeng | Gauteng | OR Tambo | Eastern Cape |
| Fezile Dabi | Free State | eThekwini | KwaZulu-Natal | Amajuba | KwaZulu-Natal |
| Mangaung | Free State | iLembe | KwaZulu-Natal | Harry Gwala | KwaZulu-Natal |
| S. Baartman | Eastern Cape | Ugu | KwaZulu-Natal | uMkhanyakude | KwaZulu-Natal |
| Johannesburg | Gauteng | uMgungundlovu | KwaZulu-Natal | uMzinyathi | KwaZulu-Natal |
| West Rand | Gauteng | uThungulu | KwaZulu-Natal | uThukela | KwaZulu-Natal |
| Nkangala | Mpumalanga | Capricorn | Limpopo | Zululand | KwaZulu-Natal |
| Frances Baard | Northern Cape | Waterberg | Limpopo | Mopani, | Limpopo |
| Namakwa | Northern Cape | G. Sibande | Mpumalanga | Sekhukhune | Limpopo |
| Pixley Ka Sema | Northern Cape | Bojanala | North West | Vhembe | Limpopo |
| ZF Mgcawu | Northern Cape | Dr. K. Kaunda | North West | Ehlanzeni | Mpumalanga |
|  |  | NM Molema | North West | RS Mompati | North West |
|  |  |  |  | JT Gaetsewe | Northern Cape |

*Note: Percentage of people living in poverty in the least poor districts: 26%-35%, middle: 35%-42%, poorest: 42%-52%.*

| *Table 9. Regression results for TB death rate + lost to follow up (DLTFU)* | | | | |  |
| --- | --- | --- | --- | --- | --- |
|  |  |  |  |  | |
| **VARIABLES** | **TB DLTFU rate** | **TB DLTFU rate** | **TB DLTFU rate** | **TB DLTFU rate** | |
| **Poverty category** | **All** | **Least poor** | **Middle** | **Poorest** | |
|  |  |  |  |  | |
| TB drug stockout proportion | -.012 | -.023 | .029 | -.045 | |
|  | (.021) | (.055) | (.039) | (.028) | |
| *Conf interval* | -.055/.030 | -.137/.090 | -.051/.108 | -.102/.012 | |
| Constant | 24.618*** | 31.741** | 29.045*** | 17.359*** | |
|  | (3.960) | (12.478) | (7.079) | (5.201) | |
| *Conf interval* | 16.754/32.481 | 5.862/57.619 | 14.544/43.546 | 6.737/27.981 | |
| District FEs | Yes | Yes | Yes | Yes | |
| Year FEs | Yes | Yes | Yes | Yes | |
| Observations | 149 | 44 | 51 | 54 | |
| Number of districts | 51 | 16 | 17 | 18 | |
| R-squared | 0.072 | 0.087 | 0.396 | 0.004 | |
| Standard errors in parentheses  *** p<0.01, ** p<0.05, * p<0.1 | | | | | |
| *Note: Analysis was controlled for TB/HIV coinfection, TB incidence and ART coverage. Districts were weighted based on the number of TB patients on treatment.* | | | | | |
